# Effects of Epidural Needle Rotation on Evolution of Unilateral Epidural Block and Patients’ Hemodynamics beside Recovery Profile in Patients Undergoing Arthroscopic Knee Surgeries; A Randomized Clinical Trial

**DOI:** 10.29252/beat-070207

**Published:** 2019-04

**Authors:** Behnam Hosseini, Faramarz Mosaffa, Shideh Dabir, Hamed Tanghatari, Mehrdad Taheri

**Affiliations:** 1 *Anesthesiology Research Center, Shahid Beheshti University of Medical Sciences, Tehran, Iran*

**Keywords:** Epidural anesthesia, Hemodynamic, Block evolution, Recovery profile, Needle rotation

## Abstract

**Objective:**

To evaluate the evolution pattern of epidural block after rotating the needle tip 45° to the operative side and evaluate its effects on patients’ hemodynamics and recovery profile in those undergoing arthroscopic knee surgery.

**Methods:**

Forty participants were randomly subdivided into control and rotation group (n=20). An 18-gauge, 3.5 inch, Tuohy needle was placed at the level of L4-5 and pushed forward into the epidural space through parasagittal approach, in control group, the needle was pushed forward to the epidural space in cephaldad 90 degrees. For the rotation group, the needle was pushed forward to the epidural space and the tip was rotated 45 degrees to the surgical side.15 mL of bupivacaine 0.5% was injected and evolution of sensory and motor blocks until 2-segment regression of the sensory level below to T10 as well as total duration of motor block and surgery were recorded. Hemodynamic parameters (HR, MAP, and SPO2), hypotension, fluid intake, vasopressors, first ambulation and spontaneous urination were recorded. Statistical analysis was performed using SPSS and P≤0.5 considered significant.

**Results:**

**S**ensory block up to T10 level, Complete motor block and time for 2-segment regression of sensory level were earlier in the 45°-rotation than in the control group (*p*<0.001). Total duration of motor block in control group was lower than rotation group (*p*<0.001).Hypotension, N&V, vasopressors and fluid intake showed no statistically difference between the two groups (*p*=0.219). First spontaneous urination and ambulation were significantly lower in rotation group (*p*<0.001).

**Conclusion:**

45 degrees’ needle rotation to the surgical side provides a faster block evolution and hastened recovery profile with no significant difference in hemodynamic fluctuations.

**Clinical trial registry:**

IRCT20130518013364N7

## Introduction

Due to increasing age of patients undergoing surgery and therefore higher co-morbidity, there has been an upsurge of interest in choice of a safe and effective technique for not only an efficient perioperative anesthesia but also for effective postoperative analgesia [1, 2]. In all surgeries and especially orthopedics, anesthesiologists attempt to employ a technique which may offer minimum physiologic changes, along with an appropriate postoperative analgesia [3-6]. 

Neuraxial blockade has a wide range of clinical applications for surgery, acute postoperative pain management, and chronic pain relief [7, 8].

Spinal, epidural, and caudal neuraxial blocks result in one or a combination of sympathetic, sensory or motor blockade depending on the dose, concentration, or volume of local anesthetic administered. Despite these similarities, there are significant technical, physiologic, and pharmacologic differences [9]. Spinal anesthesia requires a small volume of drug that is almost devoid of systemic pharmacologic effects to produce rapid (<5 minutes), profound, reproducible sensory analgesia. In contrast, epidural and caudal anesthesia progress more slowly (>20 minutes) after a large volume of local anesthetic that produces pharmacologically active systemic blood levels, which may be associated with side effects and complications unknown to spinal anesthesia [10]. Single-injection spinal or epidural anesthesia with local anesthetic is most commonly used for surgery to the lower abdomen, pelvic organs and lower limbs. Continuous catheter-based epidural infusions of dilute local anesthetics and opioids are used for postoperative pain relief after major surgery to provide analgesia for days [11].  Lumbar epidural anesthesia in humans was first described by Pages in 1921, the loss-of-resistance technique by Dogliotti in the 1930s, continuous caudal for obstetrics by Hingson in 1941, and lumbar epidural catheterization for surgery by Curbelo in 1947 [12].

More recently, however, the goals of epidural analgesia have shifted from reduction of morbidity and mortality in high-risk patients to facilitation of fast-track recovery in otherwise healthy patients undergoing various types of elective inpatient surgical procedures. Since a good post-operative pain management results in quicker recovery and earlier ambulation, in addition to fewer side effects and less hospital costs and stay. With the use of epidural anesthesia, many complications during and after surgery, including cardiovascular events, cerebral events, thromboembolic events, and possible long-term immobility will be reduced [13]. When epidural anesthesia is administered during the preoperative period, the epidural catheter is occasionally placed incorrectly and migrates into a space other than the epidural space. In other cases, although the catheter enters the epidural space, its tip deviates from the intervertebral foramen, resulting in an inadequate anesthetic effect [14]. The distribution of an epidural block cannot be controlled by gravity or patient position. Nonetheless, obtaining a preferential distribution of the epidural block towards the operative side is useful, especially when large doses of analgesics are required postoperatively to tolerate aggressive physiotherapy [15]. By evaluating the epidural catheter tip position and distribution of the injected solution by computed tomography (CT), Hogan clearly demonstrated that most epidural catheter tips are placed in an anterior or lateral position. This results in great variability in the distribution of the local anesthetic solution [16]. 

It has been reported that introducing the needle tip is positioned at an angle from the midline towards the patient's pathologic side, an effective drug spread to the target area is expected since most of the injected local anesthetic will spread to the surgical side and various reports have supported the clinical efficacy of such an intentional ‘unilateral epidural block’ [15, 16]. 

However, to our knowledge, no randomized, controlled trial have evaluated the unilateral block distribution and its probable advantages on patients’ hemodynamics and recovery profile.

The present study is designed in order to evaluate the distribution pattern of epidural block after rotating the needle tip 45° to the operative side before inserting the catheter through the needle and evaluate its effects on block evolution and patients’ hemodynamics and recovery profile in those undergoing arthroscopic knee surgery on one lower limb.

## Materials and Methods

 *Study population *

 This prospective randomized clinical trial was conducted in a tertiary medical center during 2017. Study protocol was approved by the ethics committee of Shahid Beheshti University of Medical Sciences, Tehran, Iran (IR.SBMU.REC.1396.24). The study was also registered in Iranian Registry of Clinical Trials (IRCT20130518013364N7) and written informed consent was obtained from all the participants prior the study. Study population consisted of patients scheduled for elective arthroscopic knee surgery receiving epidural block. Inclusion criteria were age <60 years and ASA physical status I/II. Patients with contraindication to central blocks, previous back surgery, as well as those with diabetes or severe cardiovascular or respiratory diseases were excluded from the study. 

 *Randomization and intervention*

 Sampling was done through block randomization and participants were randomly subdivided into control group (n=20) or rotation group (n=20). Standard monitoring (non-invasive arterial blood pressure, pulse oximetry and electrocardiography) was applied for all patients upon arrival to the operating room. Each patient received 500 ml of 9% NS intravenously before initiating the block procedure. Patients were placed in the sitting position. After sterilization and preparation of the site, the skin was anesthetized by subcutaneous injection of 3ml 2% lidocaine. Next, an 18-gauge, 3.5 inch, Tuohy needle (B-BRAUN) was placed at the level of L4-5 intervertebral space and pushed forward into the epidural space through parasagittal approach, using the loss-of-resistance technique.

- In control group, the needle was pushed forward to the epidural space in cephaldad 90 degrees and then the catheter was inserted.
- For the rotation group, the needle was pushed forward to the epidural space and the tip was rotated 45 degrees to the surgical side and then the catheter was inserted.

*The procedure and outcome measures*

  After the catheter was inserted 5 cm, the needle was removed, and the catheter was secured to the skin using tunneling technique. Then through the catheter and after negative aspiration for cerebrospinal fluid and blood, 15 mL of bupivacaine 0.5% was injected by an anesthesiologist who was blind to the patients’ group and technique of catheter insertion. Patients were placed in supine position in order to help create complete sensory and motor blocks. A blinded independent observer recorded the evolution of sensory and motor blocks on both sides every 5 min until the patient was deemed ready for surgery. The sensory block level was evaluated based on pinprick test, assessed by a verbal rating scale from 100% (normal sensation) to 0 (no sensation). 

Motor block level was evaluated using a modified Bromage score as: 

0 = no motor block; 

1 = hip blocked; 

2 = hip and knee blocked; 

3 = hip, knee, and ankle blocked

Readiness for surgery was defined as complete loss of pinprick sensation up to T10 (S1) with a modified Bromage scale ≥ 2 on the surgical side (M1).  After readiness for surgery was achieved, the evolution of sensory and motor blocks was evaluated every 15 min until 2-segment regression of the sensory level below to T10 (S2) was noted. In the case of block failure, the patient was excluded from the study. Surgery was initiated after establishing sensory and motor blocks and total duration of motor block (Bromage Score ≥2) for both sides (M2) as well as total duration of surgery (M3) were recorded. Hemodynamic parameters (HR, MAP, and SPO2) were recorded at baseline and then every 5 minutes during the first 30 min of the operation and subsequently at 15 min intervals until the end of surgery. Complications (itching, headache, hypotension, bradycardia, bleeding) were recorded in case of happening and were managed according to the protocol. Hypotension was considered to be significant if decrease in the systolic arterial blood pressure ≥ 30% from the baseline, and was treated with intravenous (IV) crystalloid infusion. If volume expansion was not effective, 10 mg IV ephedrine was administered. After the end of surgery, patients were transferred to the ward and observed for side effects such as nausea, vomiting, and urinary retention. Urinary retention was defined as the need for temporary bladder catheterization due to bladder distention and inability to urinate spontaneously. The first time that patient could raise his feet without help, as well as the first spontaneous urination were recorded in their profile. To avoid bias, all the epidural blocks were performed by the same anesthesiologist and to ensure blindness, data gathering was performed by anesthesiology residents who were blind to the patients’ group. 

 *Statistical analysis *

Sample size calculation was performed using the results of a previous study [17].

MEAN 1 = 0.7

MEAN 2 = 2.2

SIGMA 1 = 0.8

SIGMA 2 = 2.2

Sample size was estimated to be 40 (20 cases per group), considering power of 80%, and level of significance to be <0.05. Statistical analysis was performed using SPSS (v.23). Shapiro-wilk test was used for testing normality, independent sample t-test was used to compare the means of variables for two groups of cases, and Chi-Square was used to analyze categorical variables. P-value ≤0.5 considered to be statistically significant.

## Results

**Fig. 1:**
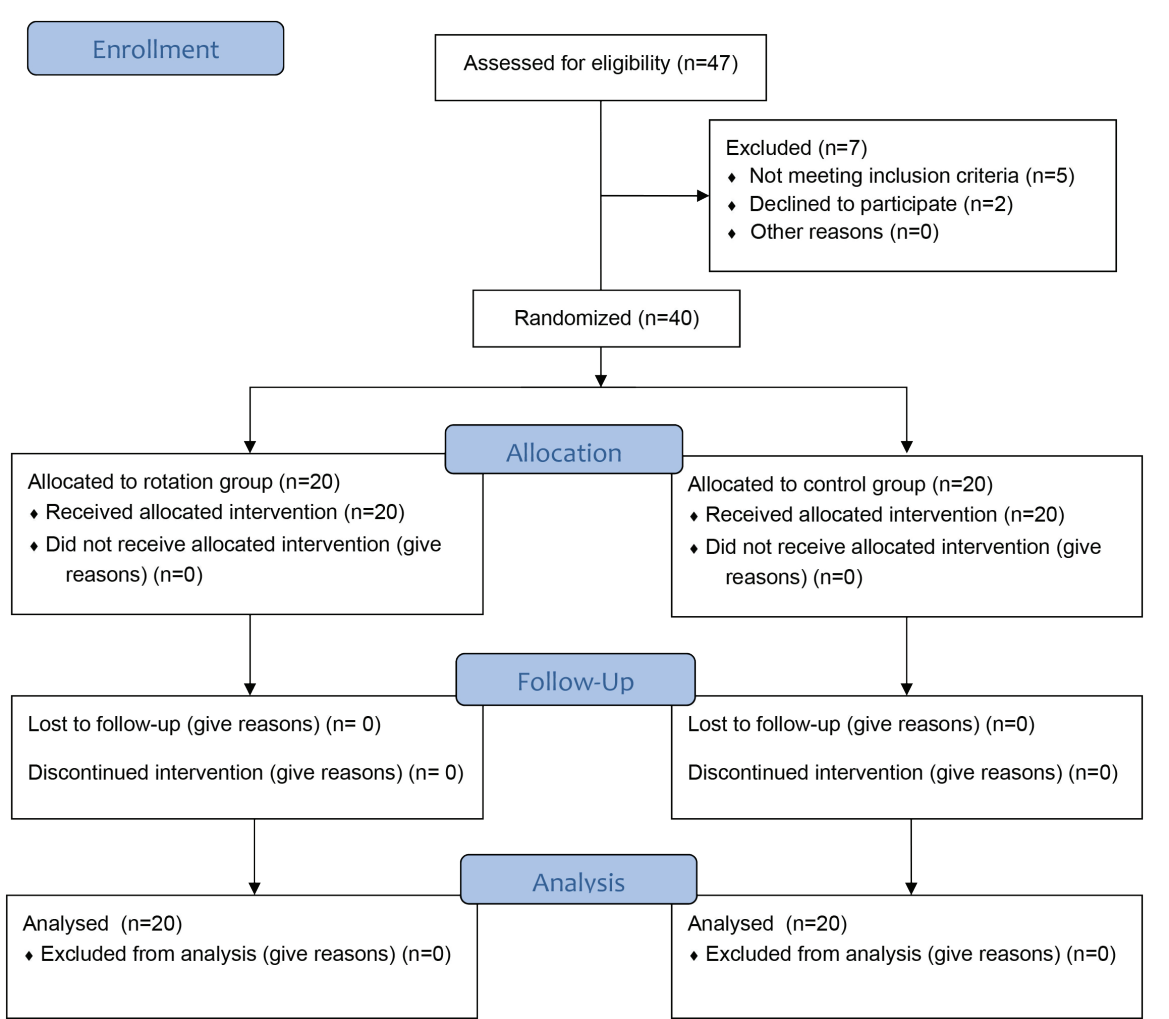
CONSORT flow diagram of the study

Forty participants completed the study (Figure 1). Using Shapiro-wilk normality test, it was shown that data were normally distributed. Regarding demographic and baseline data, there was no statistically significant difference between the two groups. Data are demonstrated in Table 1.

**Table 1:** Baseline and demographic data of patients in two groups

**Variable**	**Control Group (n=20)**	**Rotation Group (n=20)**	**p-value**
**Mean Age (yr)**	31.9 ±6.47	33.8±8.1	0.422
**Male Gender**	10	10	0.220
**BMI (m/kg2)**	24.67±3.51	24.16±4.29	0.181
**ASA (I)**	17 (85%)	17 (85%)	0.909
**ASA (II)**	3 (15%)	3 (15%)	0.511
**Duration of surgery (min)**	107.55 ± 5.44	115.5±3.02	0.083

**Fig. 2:**
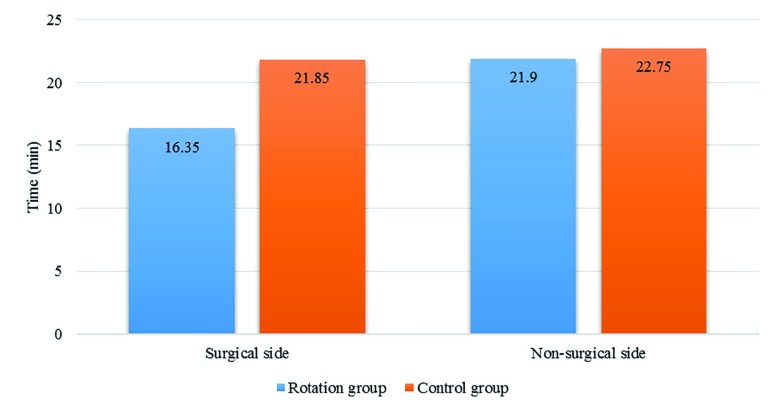
Time to achieve sensory block up to T10 in two group

In the operative foot, sensory block up to T10 level in surgical side was completed earlier in the 45°-rotation group (16.35±1.81minutes) than in the control group (21.85±2.79 minutes) and this difference was statistically significant (p=0.000). For the non-operative foot, this time showed no statistically significant difference (21.9±2.53 in control group VS 22.75±1.97 in rotation group) between the two groups (*p*=0.243). Data are demonstrated in Figure 2.

**Fig. 3:**
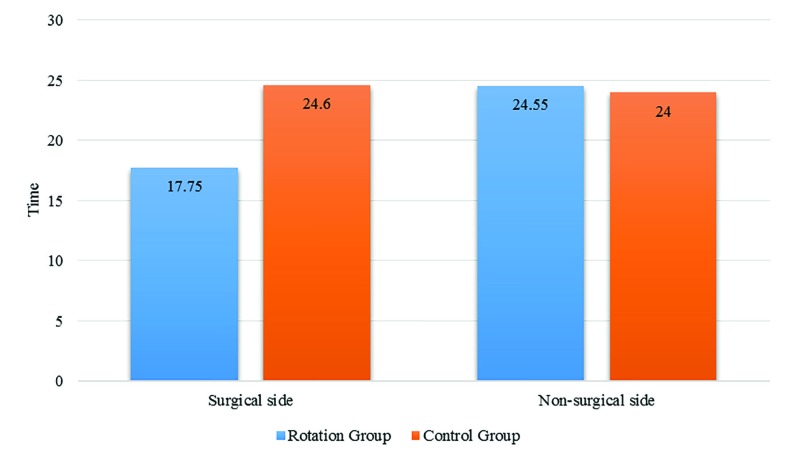
Time to achieve complete motor block in two group

Complete motor block in surgical side was achieved earlier in the 45°-rotation group (17.75±2.24 minutes) than in the control group (24.6±2.08 minutes) and this difference was statistically significant (*p*<0.001). Complete motor in non-operative side showed no significant difference (24.55±1.98 in control group *vs.* 24.00±2.05 in rotation group) between the two groups (*p*=0.394). Data are demonstrated in Figure 3.

**Fig. 4:**
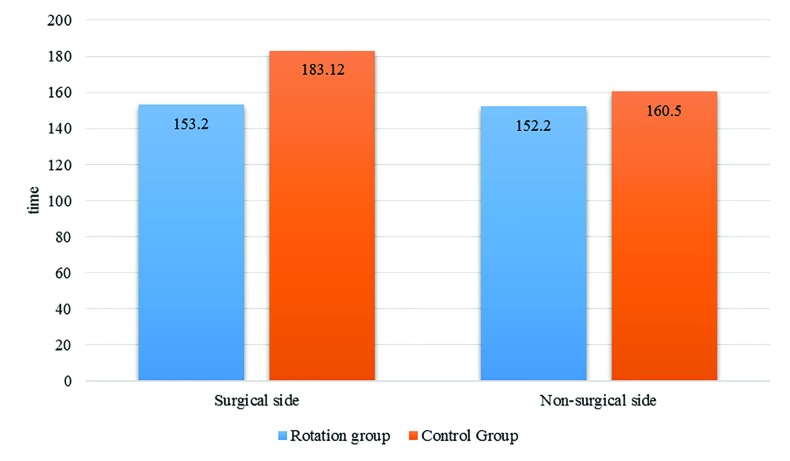
The total duration of motor block in two study groups

Total duration of motor block (Bromage Score ≥2) for operated side in control group was 153.2±11.6 minutes while this time was 183.3±12.42 minutes for rotation group. This difference between the two groups was statistically significant (*p*<0.001). In the other side, the duration of motor block in control group was 152.2±13.17 minutes and for rotation group was 160.5±11.5 minutes and this difference was also statistically significant (*p*=0.041). Data are demonstrated in Figure 4.

The time for 2-segment regression of sensory level on the operated side was 166 ± 65 minutes in the control group and 194.9±42 10.44 minutes in the rotation group and this difference was statistically significant. (*p*=0.005). However, time for 2-segment regression on the non-operative foot showed no significantly difference between the two groups, (166.7±13.75 in control group and 169.85±11.47 minutes in rotation group), (*p*=0.436). In both groups, hypotension occurred in 2 patients (10%) which was effectively treated with volume expansion. Fluid intake showed no statistically difference between the two groups (*p*=0.219). No patient required IV administration of vasopressors. Nauseas occurred in 5 patients (25%) of control group and 2 (10%) of rotation group (*p*=0.407). Vomiting was noted in 2 patients (10%) of control group, while none of the patients in rotation group developed vomiting (*p*=0.487). No severe side effects were reported in either group. Date are presented in Table 2. 

**Table 2:** Frequency of side effects in two groups

**Variable**	**Frequency (%) in Control Group (n=20)**	**Frequency (%) in Rotation Group (n=20)**	**P-value**
**Hypotension **	2 (10%)	2 (10%)	0.985
**Vasopressor**	0 (0%)	0 (0%)	0.985
**Fluid intake (L)**	1.4±0.41	1.3 ±0.29	0.219
**Nausea**	5 (25%)	2 (10%)	0.407
**Vomiting**	2 (10%)	0 (0%)	0.487

In the postoperative evaluation, the first time that patient could raise his operated foot without help, was 144.35±8.88 (min) in control group and 171.9±10.91 (min) in rotation group which proved to be statistically lower in control group (*p*<0.001). This parameter for the non-operated foot was 145.1±8.51 (min) in control group and 158.25±11.31 (min) in rotation group which also was statistically lower in control group (*p*<0.001). The first spontaneous urination was 89.8±10.52 (min) in control group and 80.1±8.38 (min) in rotation group. This parameter was significantly lower in rotation group (*p*<0.001).

## Discussion

Unilateral epidural block may occur after the placement of an epidural catheter [18]. It can however be used intentionally to provide analgesia selectively to the limb being operated. This can be achieved through rotating of the introducing Tuohy epidural needle to direct the local analgesic solution to the required side to produce an intended unilateral epidural block [15, 17]. Producing a preferential distribution of the epidural block toward the operative side may have potential advantages in patients undergoing unilateral surgery on the lower limb. In the present study, the epidural needle tip rotation for 45 degrees toward the surgical side was associated with a faster sensory block in comparison with the conventional method of cephaldad 90 degrees. Complete motor block in surgical side was achieved earlier in the 45°-rotation group and total duration of motor block (Bromage Score ≥2) and the time for 2-segment regression of sensory block was statistically longer than the control group. 

Intentional unilateral blocks have been previously described in several case reports. Buchheit et al., [19] in 2000, reported a case of a woman with a history of complex regional pain syndrome of the right upper extremity. They placed an epidural catheter at the C6-7 vertebral interspace and directed the needle bevel and catheter to the affected side and realized that laterally directed cervical epidural catheter was an effective technique to produce continuous unilateral analgesia and sympathetic block. Fukishige *et al*., reported a case of unilateral epidural block developing after each of three attempts at single injection epidural block and discuss the cause of unilateral epidural block based on radiographic findings. They suppose that the epidural needle was placed in the left posterior epidural space and that the presence of dorsomedian connective tissue and deformation of the dural sac to a contracted inverted triangle after epidural injection caused the distension of the left posterior epidural space and prevented the spread of local anesthetics to the right side of the epidural space which seemed to be responsible for unilateral epidural block [20].Several studies have evaluated the effects of needle tip position on distribution patterns of anesthetic solutions and its consequent effects on uniform epidural anesthesia. In a randomized clinical trial conducted by in 2004, Borghi *et al*. evaluated the effects of turning the tip of the Tuohy needle 45 degrees toward the operative side before threading the epidural catheter as compared to a conventional insertion technique with the tip of the Tuohy needle oriented at 90 degrees cephalad on distribution of anesthetic agent in patients undergoing total hip replacement. Findings from their trial showed that this simple maneuver provides a preferential distribution of sensory and motor block toward the operative side which is consistent with findings from the present study [17]. Despite the literature supporting the hypothesis, few inconsistent studies do exist. Kwon et al in 2016 tested the hypothesis that the needle position or bevel direction relates to the pattern of epidural spread during CESI, although their findings revealed that neither the needle tip position nor did the bevel direction affect the epidural drug spreading pattern during CESI. They believed  that with no correlation in 210 cases, their hypothesized relation between needle tip position or bevel direction and epidural spreading pattern that was tested in this study is likely not useful in clinical cases [21].

In patients with chronic lower extremity radiculopathy, segmental nerve root blocks (SNRBs) are performed to putative symptomatic spinal nerve. In a 2006 study, authors assessed epidural local anesthetic spread and its relationship to needle position during fluoroscopy-assisted blocks. 

Patients scheduled for L4, L5, and S1 blocks were included. Epidural spread occurred more frequently with medial needle positions. The findings suggest that the risk of grade 1 and 2 lumbar epidural spread, which results in decreased SNRB selectivity, is greater with medial needle positions in the intervertebral foramen [22]. As an invasive technique, epidural anesthesia’s benefit/risk ratio deserve to be appraised in order to help the physicians to make the appropriate choice among other opportunities.  In our study, regarding patients’’ hemodynamic profile, the occurrence of hypotension was equal in both groups which was successfully managed by IV fluids and none of the patients required administration of vasopressors. Nauseas and vomiting were also noted almost equally in both groups. Regarding patients’ recovery profile in postoperative evaluation, the first spontaneous urination was sooner in rotation group. These findings reveal that this simple discussed maneuver has no significant cardiovascular or respiratory effect on patients’ hemodynamic and can be safely used for a better recovery profile in patients undergoing lower extremity orthopedic surgeries.

In this study, the potential effect of epidural needle rotation and consequent increased concentration of anesthetic solution in the operative side and its probable effects on post-operative pain intensity is not evaluated. Further studies can be conducted to evaluate any potential reduction of pain intensity in post-operative period. In addition, the routes of distribution and barriers to flow of solutions in the epidural space, specific degree of sympathetic block and/or total volume of anesthetic requirement for block evolution may be considered for further studies.

In conclusion, findings of the present study revealed that 45 degrees’ rotation of Tuohy needle toward the surgical side before insertion of the epidural catheter provides a faster evolution of sensory and motor blocks in the operative side, and hastened recovery profile with no significant difference in hemodynamic fluctuations.

## Conflict of Interest:

None declared.
